# Effects of light on brain-state dynamics and energy landscape

**DOI:** 10.1162/NETN.a.586

**Published:** 2026-08-25

**Authors:** Rui Zhang, Nora D. Volkow

**Affiliations:** Department of Psychiatry and Behavioral Neurobiology, University of Alabama at Birmingham, Birmingham, AL, USA; National Institute on Drug Abuse, National Institutes of Health, Bethesda, MD, USA

**Keywords:** Light intensity, Brain-state dynamics, Control energy, Network control theory

## Abstract

Light influences human cognition and behavior, and neuroimaging studies show that brain activity is modulated by light intensity. However, how light affects temporal brain-state transitions and the control energy required for these transitions remains unclear. To investigate this, we applied a network control theory approach to fMRI data collected from 20 healthy participants who performed an auditory discrimination task under four light intensities. Despite similar task performance, higher light intensity increased the number of transitions between brain states. Increasing light intensity enhanced the occurrence of a visual network dominated brain state, while decreasing the occurrence of brain states characterized by suppressed default mode activity and elevated frontoparietal activity. Furthermore, light intensity affected transition probabilities among different brain states contributed by redistributions of energy demands with the dorso-posterior thalamus appearing to play a key role in mediating light-related effects. Regionally, higher light intensity was associated with a trend toward reduced control energy in the visual network; frontal, cingulate, and insular cortices; and caudate and task-related regions, while showing a trend toward increased control energy in the somatomotor network and temporal pole. These findings suggest that high-intensity light may enhance neural efficiency and flexibility by redistributing control energy demands across brain regions.

## Author Summary

Light regulates human mood, cognition, and behavior. The current study investigated how light intensity influences brain-state dynamics and the control energy underlying these changes using network control theory. Higher light intensity increased the frequency of brain-state transitions, indicating enhanced neural flexibility. Exposure to brighter light also promoted a brain state dominated by visual network activity, while reducing the occurrence of states characterized by suppressed default mode network activity and elevated frontoparietal engagement. Moreover, light intensity altered transition probabilities between brain states through a redistribution of control energy across brain regions. Control energy mediated the effects of light on brain-state dynamics, with the dorso-posterior thalamus emerging as a central mediator.

## INTRODUCTION

Light modulates cognition and well-being in humans (Mahoney & Schmidt, 2024). Laboratory studies have documented the effect of acute light exposure on working memory (Vandewalle et al., 2007), attention, and alertness (Chellappa et al., 2011). Light exposure intervention has been shown to reduce daytime sleepiness and improve executive function in patients recovering from mild traumatic brain injury (Killgore, Vanuk, et al., 2020) and among people living with dementia, although the effects of light on cognition were smaller than those observed for mood-related symptoms (van Maanen et al., 2016). Neuroimaging studies indicate that light particularly blue light, modulates activation and connectivity in frontoparietal regions involved in executive function and subcortical regions involved in arousal, memory, and emotion (Alkozei et al., 2016; Killgore, Dailey, et al., 2020; Vandewalle et al., 2007, 2010).

Beyond static measures of brain activity and connectivity, the assessment of temporal brain dynamics is crucial for understanding brain function. Dynamic brain markers reveal insights about clinical symptoms and cognitive impairments that are not captured by static brain measures based on averaged activity (Nguyen et al., 2017; Rashid et al., 2016). Greater brain variability and state transitions, both during tasks and rest, have been associated with better cognitive performance (Garrett et al., 2011; Nguyen et al., 2017). Conversely, patients with attention-deficit/hyperactivity disorder (ADHD), bipolar disorder, and opioid use disorder have decreased brain-state transitions and variability associated with cognitive impairment (Nguyen et al., 2017; Sun et al., 2021; Ye et al., 2024). Despite these findings, it remains unknown whether and how light influences brain-state transitions and dynamics. In the current study, rather than identifying regions exhibiting transient or sustained response to various light intensities, as in Sabbah et al. (2022), we focused on temporal brain-state dynamics at the network level and the energy required for transitions between brain-states. This approach provides temporal, functional, and metabolic insights into how light intensity shapes large-scale brain networks.

Light influences brain function through rods and cones, which mediate image-forming vision, and intrinsically photosensitive retinal ganglion cells (ipRGCs), which primarily support nonimaging forming functions such as circadian rhythms and mood regulation. In animals, ipRGCs project to and modulate brain regions that are largely distinct from those targeted by rods and cones including prefrontal and associative cortices, reflecting their role in nonvisual processing (Fernandez et al., 2018; Lazzerini Ospri et al., 2024). Unlike rods and cones with fast and transient responses to light, ipRGCs exhibit slower and more sustained responses, although they also integrate synaptic inputs from rods and cones (Berson et al., 2002). Consequently, prolonged light exposure is expected to alter brain dynamics in ways that depends on the relative contributions and temporal properties of ipRGC and rod/cone activity, specifically, rods and cones are expected to contribute more strongly during the initial phase of light exposure, whereas ipRGCs are expected to dominate during later phases of light exposure. We therefore anticipated that brain-state dynamics would differ between the first half and the second half of light exposure. Light may trigger immediate sensory responses in the visual network (VIS) through direct retinal inputs primarily from rods and cones. With prolonged exposure, the brain may begin to integrate this sensory information into broader attention and cognitive control system, engaging associative cortices to support sustained attention for tasks. Additionally, ipRGC-mediated influences on the brain networks involving prefrontal and associative regions may become more pronounced during the later phase of light exposure. Specifically, we expected that a VIS-dominated brain state would occur more frequently during the initial phase of light exposure, whereas a brain state dominated by associative cortices such as the frontoparietal network (FPN) and default mode network (DMN) would become more prominent later with extended light exposure.

Furthermore, we investigated whether light-induced changes in brain-state transitions are driven by altered control energy demanded for transitions. Our previous work revealed regions in which brain glucose metabolism was associated with seasonal changes in light exposure (Zhang et al., 2024). Regions that are more active and exhibit higher metabolic activity in summer may show elevated activity under higher light intensity and require lower control energy to drive brain-state transitions.

Here, we applied network control theory (NCT), a mathematic tool that models brain-state dynamics as a function of brain structure and control energy and calculates the minimum energy needed for transition between different brain states or persisting in the same brain state based on normative structural connectome data obtained from diffusion MRI (Gu et al., 2015; Kim et al., 2018). As the dorso-posterior thalamus (thalamus_DP_) plays an important role in relaying light inputs from retina to cortex including the visual, prefrontal and associative regions (Paparella et al., 2023; Weil et al., 2022), we compared thalamus_DP_-only versus whole-brain control inputs in NCT to test whether the visual thalamus primarily drives light-induced changes in control energy underlying brain dynamics.

## MATERIALS AND METHODS

### Participants and fMRI Experimental Design

Twenty healthy participants took part in the study (10 females; mean age ± *SD*: 24.3 ± 3.7 years) and provided written informed consent. Functional MRI data were acquired using a 3T Siemens Prisma scanner (2-mm isotropic voxels, repetition time [TR]: 2 s, echo time [TE]: 30 ms, field of view [FOV]: 212 mm) to examine the whole-brain activation patterns in response to full-field diffused white light stimuli. To maintain alertness, participants were asked to perform an auditory task of discriminating between two tones presented in pseudorandom order and to keep their eyes open throughout the scan. The session included five runs, each lasting 6 min. Each run consisted of twelve 30-s epochs of consistent light intensity. Four light intensities were presented in pseudorandom order (mean irradiance across the 400- to 700-nm human visible spectrum L1: 10.2, L2: 12.1, L3: 13.1, and L4: 13.8 log photons cm^−2^ s^−1^). Each intensity, that is, L1–L4 was presented three times per run, resulting in a total of 15 epochs per light intensity across the five runs (Supporting Information Figure S1). This study was approved by the Brown University institutional review board. The imaging data are available from an open-source repository: doi:10.18112/openneuro.ds004065.v1.0.0. Behavioral data from the auditory task are not publicly available but are reported in Sabbah et al. (2022), where no significant effects of light intensity on task performance were observed (Sabbah et al., 2022).

### MRI Preprocessing

The data were preprocessed using CONN toolbox 22a (Whitfield-Gabrieli & Nieto-Castanon, 2012), including slice timing, rigid body realignment, spatial normalization to Montreal Neurological Institute space, smoothing (full width at half maximum = 4 mm), bandpass filtering (0.01–0.08 Hz), linear detrending, head motion regression (three rotational, three translational, and their derivatives), and removal of signals within the cerebrospinal fluid and the white matter (WM) using aCompcor, a method for identifying principal components associated with segmented WM and cerebrospinal fluid.

### Analysis of Brain States and Dynamics

To identify brain states, that is, brain coactivation patterns, denoised voxel-level data (percent signal changes) were first parcellated into a 454-node Schaefer atlas (400 cortical regions and 54 subcortical regions) (Schaefer et al., 2018; Tian et al., 2020), and each ROI time series was demeaned. Then, we concatenated demeaned regions of interest (ROI) time series from all participants, all light conditions and all runs and applied *k*-means clustering using Pearson correlation as the distance metric (Cornblath et al., 2020). This approach allowed us to investigate brain dynamics with the maximal temporal resolution of 1 TR. In this study, each subject had in total 900 TRs across all light conditions and five runs. We performed *k*-means clustering for *k* = 2–30, where *k* was the number of clusters (*k*^2^ must be less than the number of TRs to capture all transitions per subject) (Cornblath et al., 2020) and repeated 50 times with the random initializations before choosing the solution with the best data separation. The optimal number of clusters *k* = 5 was chosen because the additional variance explained by increasing *k* beyond 5 was less than 1% (Supporting Information Figure S2). Clusters were defined as brain states and labeled by assessing the cosine similarity of the positive and negative activations of their centroid with a binary presentation of seven a priori-defined cortical brain functional networks (Yeo et al., 2011) plus a network with 54 subcortical regions. Given a TR = 2 s, each 30-s epoch of a consistent light intensity comprised 15 TRs (Supporting Information Figure S1). We defined the first 7 TRs as the first half of the epoch and the remaining 8 TRs as the second half. For each half, we calculated fractional occupancy (FO) defined as the proportion of TRs assigned to each brain state. Additionally, we computed the total number of brain-state transitions for each light intensity as well as transition probability between brain states i and j defined as the probability that state j occurs at the TR after state i, given that state i is occurring. Due to the limited number of transitions (up to 14 per 30-s epoch), we did not examine total number of brain-state transitions and transition probability between brain states for the first half versus second half of the epoch separately.

### Structural Connectivity Network Construction

A group-average template was constructed from a total of 1,065 scans. Multishell diffusion MRI was acquired using *b*-values of 1,000, 2,000, 3,000 s/mm^2^, each with 90 directions and 1.25 mm iso-voxel resolution. The diffusion data were reconstructed in the MNI space using q-space diffeomorphic reconstruction (Yeh et al., 2011) to obtain the spin distribution function (Yeh et al., 2010). The restricted diffusion was quantified using restricted diffusion imaging (Yeh et al., 2017). A deterministic fiber tracking algorithm (Yeh et al., 2013) was applied with augmented tracking strategies (Yeh, 2020) to improve reproducibility using parameters: anisotropy threshold randomly between 0.5 and 0.7 Otsu threshold, angular threshold = 55 degrees, step size = 1.00 mm, and track length between 10 and 400 mm. A total of 1,000,000 tracts were generated. 454 ROIs were used for the brain parcellation. Connectivity matrix was calculated by using count of the connecting tracks. Lastly, streamline count was normalized by the number of voxels contained in each pair of ROIs.

### Control Energy Calculations

NCT advances our understanding of how WM structure constrains brain dynamics and allows us to compute minimum energy required for brain-state transitions and persistence given the WM connections (Cornblath et al., 2020). While similar procedures has been described elsewhere (Cornblath et al., 2020; Singleton et al., 2022; Zhang et al., 2025), we summarize them briefly here. We employ a linear time-invariant model:

$$\dot{\text{x}}\left(\text{t}\right)=\text{Ax}\left(\text{t}\right)+\text{Bu}\left(\text{t}\right)$$ (1)

where $\dot{\text{x}}$ is the brain regional activity at time t; A represents N × N structural connectivity matrix; and *N* is the number of brain regions, that is, *N* = 454 in this study. B contains the control input weights for each brain region, that is, how much energy can be injected into a specific brain region; and u is the control energy (external input) being injected over time. In this study, we calculated minimum control energy under two conditions where the B matrix varied. Model 1 (whole-brain control inputs): The identity matrix was used for uniform control (diagonal values in B identity matrix were 1). Model 2 (Thalamus_DP_-only control inputs): Diagonal values in B matrix were 1 for left and right dorso-posterior thalamus and 0 s elsewhere, that is, no inputs were allowed to other regions. To compute the minimum control energy required to drive the system from an initial brain state x_0_ to a target state x_T_ over T, we computed an invertible controllability Gramian for controlling the network A from N nodes.

$$W=\int_{0}^{T}{\text{e}}^{\text{A}\left(T-\tau \right)}\;\text{B}{\text{B}}^{\text{T}}{\text{e}}^{{\text{A}}^{\text{T}}\left(T-\tau \right)}\;\text{d}\tau$$ (2)

The minimum control energy E_m_ was computed as the quadratic product between the inverted controllability Gramian and the difference between x_0_ and x_T_. For persistence energy, x_0_ = x_T_.

$${\text{E}}_{\text{m}}={\left({\text{e}}^{AT}{\text{x}}_{0}-{\text{x}}_{\text{T}}\right)}^{\text{T}}{\text{W}}^{-1}\left({\text{e}}^{AT}{\text{x}}_{0}-{\text{x}}_{\text{T}}\right)$$ (3)

To calculate the E_m_, time horizon *T*, which defines the time over which input to the system is allowed, needs to be specified. When T approaches 0, E_m_ calculation is less affected by structural connectome A. With longer T, both the brain state space distance between initial and final state in the transition and the topology of A determine E_m_. We selected a time horizon of T = 1, which is a common choice (Parkes et al., 2023).

$${\text{A}}_{\text{norm}}=\frac{A}{{\left|\lambda \left(A\right)\right|}_{\max}+c}-I$$ (4)

To normalize the adjacency matrix A, we set c = 1 by default to ensure the activity goes to zero over time that is necessary for the stabilization of the system. |*λ*(*A*)|_*max*_ denotes the largest absolute eigenvalue of the system. *I* denotes the NxN identity matrix.

For each subject, we computed the control energy required for the whole brain to transition between each pair of brain states (pairwise global control energy) and then averaged across all state pairs to obtain mean global control energy. We also calculated mean regional control energy by averaging the control energy required for all transitions between five brain states (i.e., the mean of pairwise regional control energy), separately for each light condition (Singleton et al., 2023).

### Statistical Analyses

Two-way repeated-measures analyses of variance were conducted to examine the effects of light intensity (four levels) and duration of light exposure (first half vs. second half), as well as their interaction, on the FO of brain states.

Linear mixed-effects models were used to examine the effects of light intensity on the number of brain-state transitions, transition probabilities between different brain states, and control energy (mean global control energy, pairwise control energy, and mean regional control energy). Mean light irradiance was included as a continuous predictor with a random intercept for each subject. Multiple comparisons were corrected using the Benjamini–Hochberg procedure across tests involving five brain states, 20 possible brain-state transitions, or 454 regions of interest. Associations between control energy and transition counts or probabilities were accessed using Pearson correlation coefficients.

Mediation analyses were used to examine whether control energy mediated the effect of light intensity on brain-state transitions. They were conducted only when the effects of light intensity on transitions, the effect of light on control energy, and the association between brain-state transitions and control energy were all significant. The significance of the indirect effect was assessed using a bootstrap procedure with 5,000 resamples to generate 95% confidence intervals, with mediation considered significant if the interval did not include zero.

## RESULTS

### Brain States

**Figure 1.**
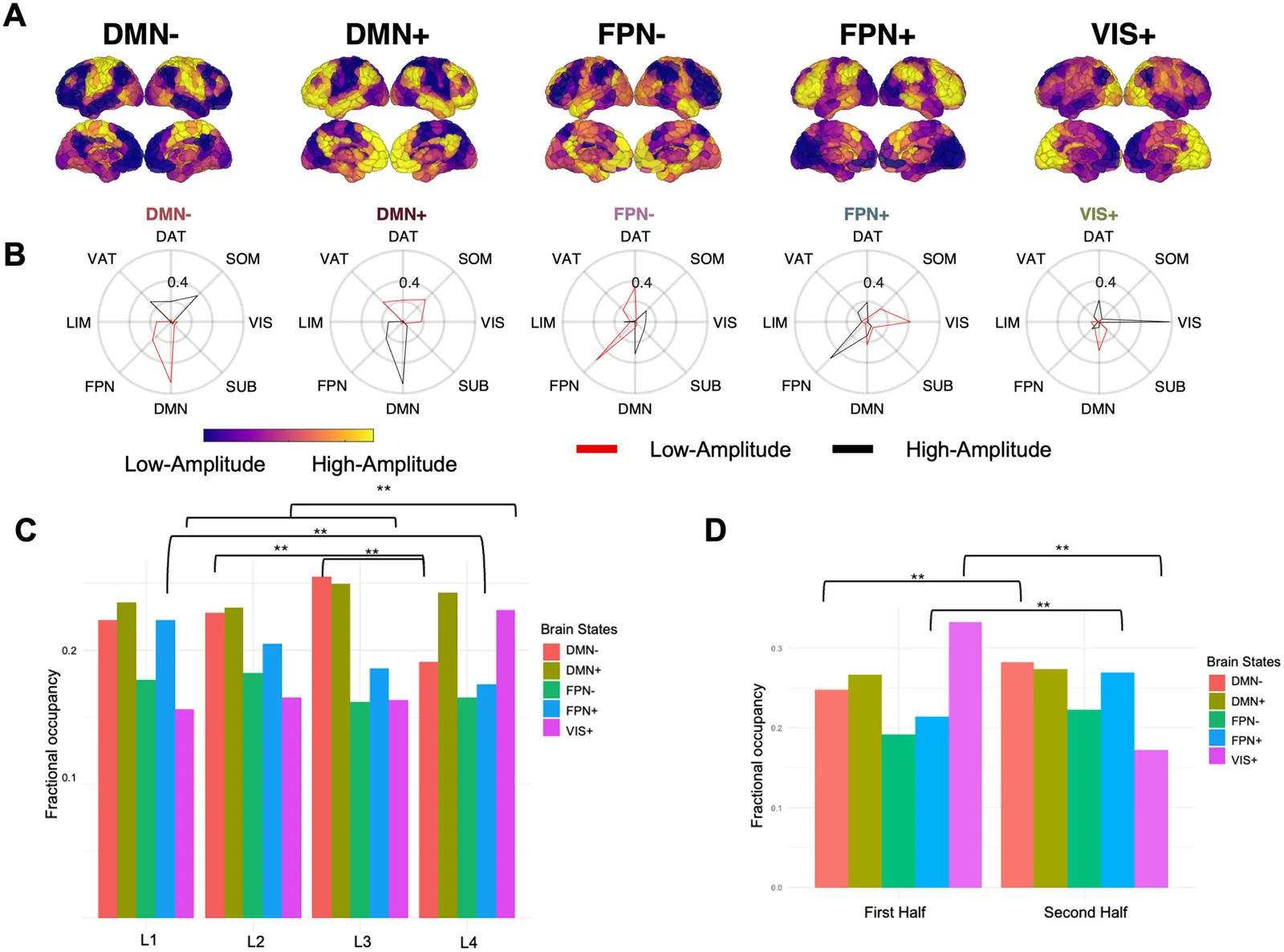
Effects of light intensity and exposure duration on brain-state dynamics. (A) Brain states were identified using a data-driven approach and labeled based on the cosine similarity with a priori-defined resting-state functional networks. Anatomical representation of brain states with their centroids mapped to colors of the corresponding 454 regions of the Schaefer atlas consisting of 400 cortical and 54 subcortical regions. Centroids of each state were calculated as the mean of the regional activation over all TRs assigned to that state. The label reflects resting-state functional networks with the most overall similarity. (+)/(−) represents activity above/below regional means. (B) Radial plot of each brain state represents cosine similarity of its high-amplitude and low-amplitude activity with resting-state functional networks. Larger values correspond to higher similarity. DAT = dorsal attention network, DMN = default mode network, FPN = frontoparietal network, LIM = limbic network, SOM = somatomotor network, VAT = ventral attention network, VIS = visual network, SUB = subcortical regions. (C) Main effect of light intensity (averaged across the first and second halves). (D) Main effect of exposure duration (first vs. second half, averaged across light conditions) on FO of five identified brain states. **p_corrected_ < 0.05.

We identified five recurrent coactivation patterns referred to as brain states. They were highly similar under different light intensities (Supporting Information Figure S3). Based on the predominance of the network patterns in each brain state, the five brain states were labeled as default mode (DMN+, DMN−), frontoparietal (FPN+, FPN−), and visual (VIS+), with + and − indicating activity above or below regional means, respectively (Figures 1A and 1B).

### Main Effects of Light Intensity on Brain-State Dynamics

Light intensity significantly affected the FO of FPN+ (*F* = 4.155, *p* = 0.010, *p*_corrected_ = 0.050, partial *η*^2^ = 0.179), VIS+ (*F* = 9.416, *p* < 0.001, *p*_corrected_ = 0.005, partial *η*^2^ = 0.331) and DMN− (*F* = 7.327, *p* < 0.001, *p*_corrected_ = 0.005, partial *η*^2^ = 0.278). Under the highest light intensity condition (L4: 13.8 log photons cm^−2^ s^−1^), FPN+ FO was lower than under the lowest light intensity condition (L1: 10.2 log photons cm^−2^ s^−1^; post hoc: mean difference = −0.047, *p*_corrected_ = 0.017). DMN– FO was lower under the L4 condition compared to under the L2 (12.1 log photons cm^−2^ s^−1^) and L3 (13.1 log photons cm^−2^ s^−1^) conditions (post hoc: all mean differences > 0.037, all *p*_corrected_ < 0.047). In contrast, VIS+ FO was higher under L4 than under all other three lower intensity conditions (post hoc: all mean differences > 0.054, all *p*_corrected_ < 0.005; Figure 1C and Supporting Information Figure S4).

### Main Effects of Light Exposure Length (First Half vs. Second Half) on Brain-State Dynamics

FPN+ FO and DMN− FO was higher in the second half than in the first half of light exposure (FPN+: mean difference = 0.036, *F* = 16.919, *p* < 0.001, *p*_corrected_ = 0.005, partial *η*^2^ = 0.471; DMN−: mean difference = 0.062; *F* = 45.408, *p* < 0.001, *p*_corrected_ = 0.005, partial *η*^2^ = 0.705), while VIS+ FO was higher in the first half than the second half (mean difference = 0.136, *F* = 55.359, *p* < 0.001, *p*_corrected_ = 0.005, partial *η*^2^ = 0.744; Figure 1D and Supporting Information Figure S4).

### Interaction Effects of Intensity and Length of Light Exposure on Brain-State Dynamics

We did not observe significant interaction effects between light intensity and the exposure length on brain-state dynamics (all *p* < .113; Supporting Information Figure S4).

### Effects of Light Intensity on The Number of Brain-State Transitions and Transition Probabilities Between Different Brain States

**Figure 2.**
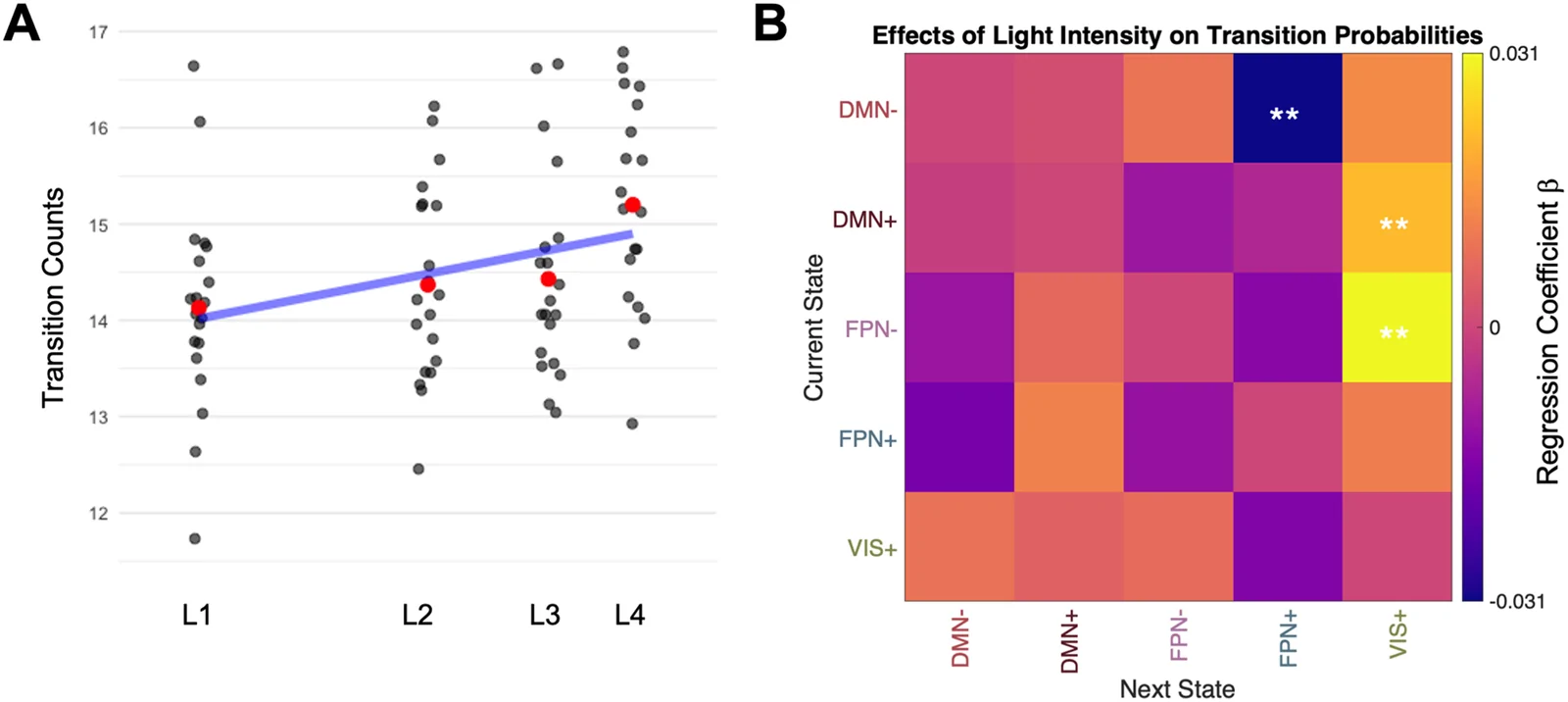
Effects of light intensity on the number of brain-state transitions, transition probabilities between different brain states. (A) Higher light intensity was associated with an increased number of brain-state transitions. L1: 10.2, L2: 12.1, L3: 13.1, L4: 13.8 log photons cm^−2^ s^−1^. Each point represents the average number of transitions per subject across runs. Each 6-min run comprises 180 TRs and is divided into four light conditions resulting in up to 44 brains state transitions per condition. (B) Transition probabilities between brain states varied as a function of light intensity. Regression coefficients from the linear mixed-effects models are shown. **p_corrected_ < 0.05, *p_uncorrected_ < 0.05.

The total number of brain-state transitions increased with light intensities (*β* = 0.245, *p* = 0.002; Figure 2A). Regarding transition probabilities between different brain states, light intensity significantly decreased brain-state transition probability from DMN− to FPN+ (*β* = −0.030, *p* = 0.003, *p*_corrected_ = 0.027) and increased transition probability from DMN+, FPN− to VIS+ (all *β* > 0.021, all *p* < 0.004, all *p*_corrected_ = 0.027; Figure 2B).

### Effects of Light Intensity On Global and Regional Control Energy

#### Global control energy.

##### Model 1 (Whole-Brain Control Inputs).

**Figure 3.**
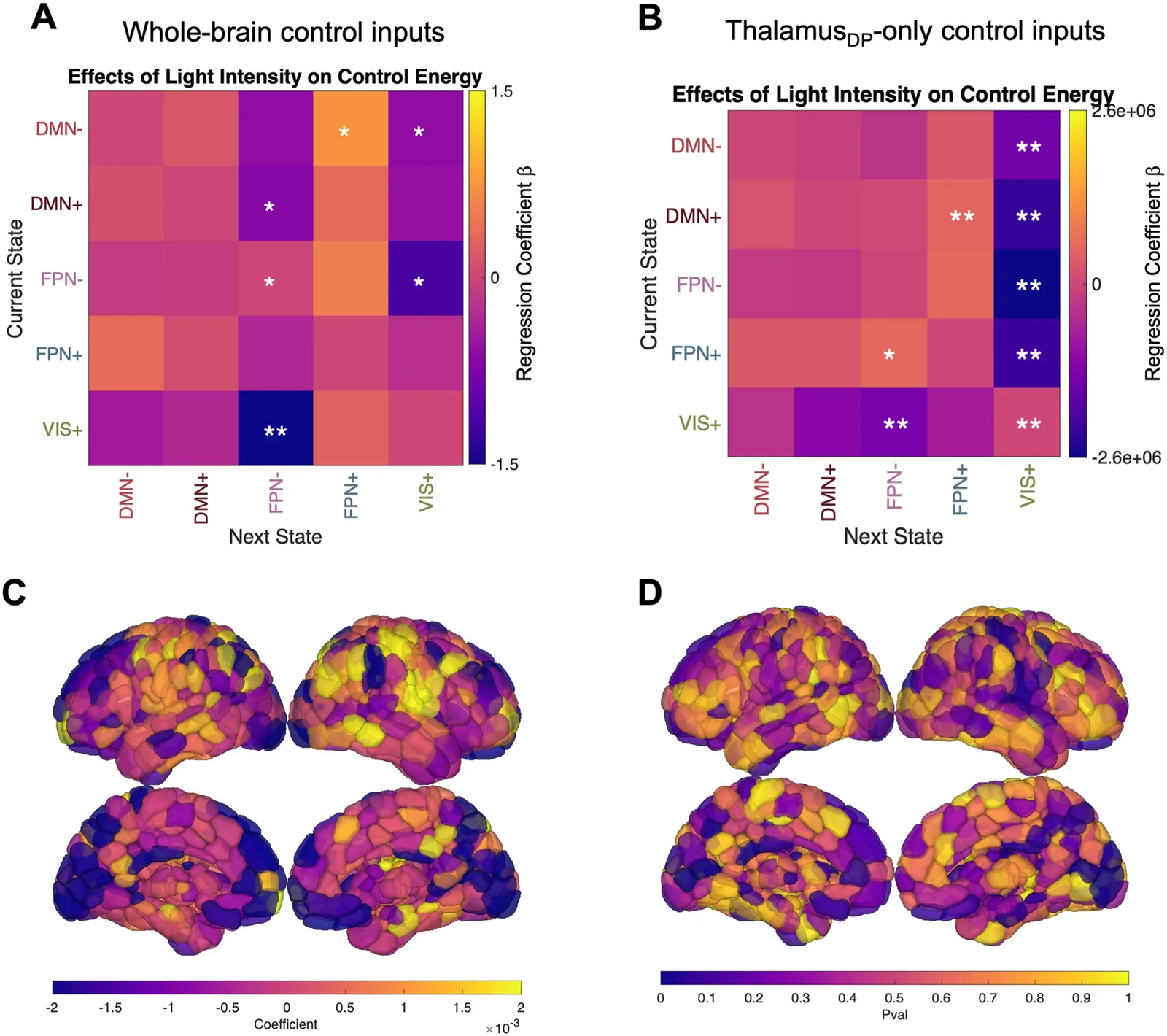
Effects of light intensity on control energy. Control energy required for transition between brain states varied as a function of light intensity. (A) Model 1: whole-brain control inputs. (B) Model 2: thalamus-only control inputs. Regression coefficients from the linear mixed-effects models are shown. **p_corrected_ < 0.05, *p_uncorrected_ < 0.05. (C) Mean regional control energy varied as a function of light intensity. Regression coefficients are shown. (D) Corresponding *p*_uncorrected_ values are displayed.

For mean global control energy, the effect of light intensity was not significant (*β* = −0.164, *p* = 0.385). For pairwise global control energy, higher light intensity significantly reduced the energy required for transitioning from the VIS+ to the FPN− state (*β* = −1.51, *p* < 0.001, *p*_corrected_ = 0.011). Additionally, there was a trend toward reduced energy demand for transitions from DMN− and FPN− to VIS+ (all *β* < −0.610, all *p* < 0.033, all *p*_corrected_ < 0.137), from DMN+ to FPN− (*β* = −0.675, *p* = 0.029, *p*_corrected_ = 0.137), and for persisting in the FPN− state (*β* = −0.266, *p* = 0.019, *p*_corrected_ = 0.137). In contrast, higher light intensity was associated with increased control energy required for transitions from DMN− to FPN+ (*β* = 0.744, *p* = 0.033, *p*_corrected_ = 0.137) (Figure 3A).

##### Model 2 (Thalamus_DP_-Only Control Inputs).

For mean global control energy, higher light intensity was associated with lower mean global energy (*β* = −2.14e+10, *p* = 0.049). For pairwise global control energy, higher light intensity significantly reduced the control energy required for transitions to the VIS+ state (all *β* < −7.58e+10, all *p* < 0.011, all *p*_corrected_ < 0.039) and from the VIS+ to the FPN− state (*β* = −7.14e+10, *p* = 0.003, *p*_corrected_ = 0.019). In contrast, higher light intensity was associated with increased control energy required for transitions from DMN+ to FPN+ (*β* = 2.69e+10, *p* < 0.001, *p*_corrected_ = 0.010) and a trend from FPN+ to FPN− (*β* = 3.13e+10, *p* = 0.039, *p*_corrected_ = 0.122) (Figure 3B).

#### Regional control energy (whole-brain control inputs).

At regional level, higher light intensity was associated with a trend toward reduced control energy for brain-state transitions in the VIS, as well as in middle and orbitofrontal gyrus, anterior and posterior cingulate cortices, insula, caudate, and superior temporal gyrus, while showing a trend toward increased control energy in the somatomotor network (SOM) and temporal pole (Figures 3C and 3D, Supporting Information Table S1).

### Associations Between Control Energy and Transition Counts/Probabilities

#### Model 1 (whole-brain control inputs).

Across light conditions, the association between mean global energy and total transition counts was not significant (*r*_80_ = −0.11, *p* = 0.338).

**Figure 4.**
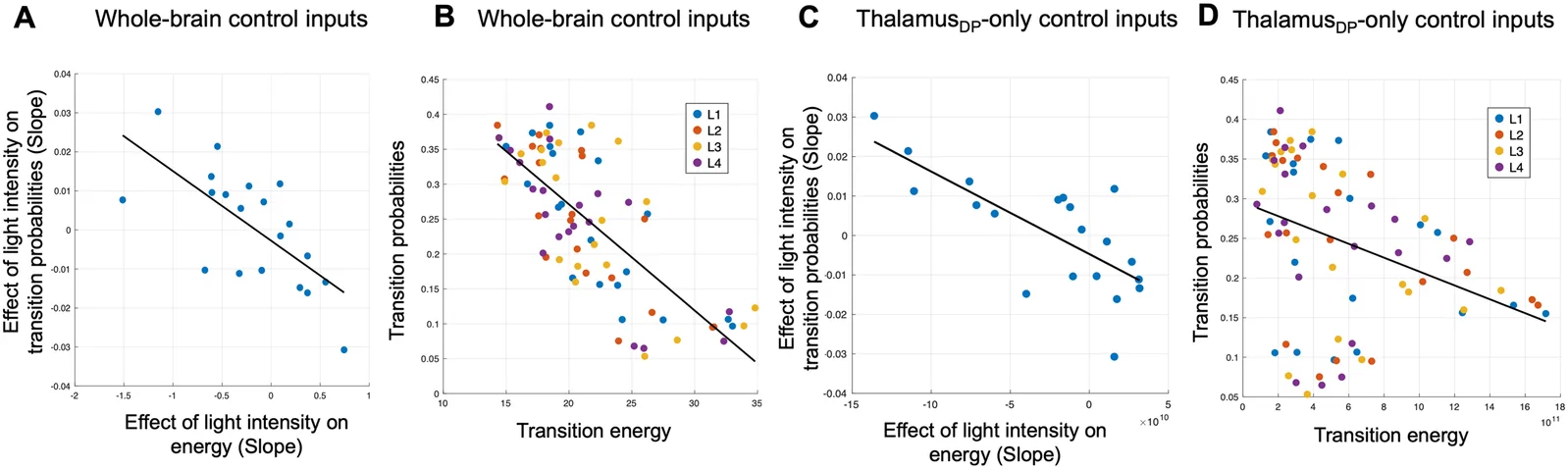
Associations between control energy and transition probabilities. (A & C) Association of the effect of light on transition probabilities (i.e., regression coefficients/slope) and its effect on transition energy (i.e., regression coefficients/slope; control energies for 20 transition pairs are included). (B & D) A negative association between transition energy and transition probabilities of brain states (20 brain-state transition pairs across four light conditions).

The effect of light on transition energy (i.e., regression coefficients) was negatively associated with its effect on transition probabilities (20 transition pairs; *r*_20_ = −0.69, *p* < 0.001; Figure 4A). Across light conditions, brain-state transitions that require less control energy had higher transition probabilities (*r*_80_ = −0.75, *p* < 0.001; Figure 4B).

#### Model 2 (Thalamus_DP_-only control inputs).

Across light conditions, there was a negative association between mean global energy and total transition counts (*r*_80_ = −0.30, *p* = 0.007). The effect of light on transition energy was negatively associated with its effect on transition probabilities (*r*_20_ = −0.74, *p* < 0.001; Figure 4C). Across light conditions, transitions between brain states that required less control energy occurred more frequently (*r*_80_ = −0.36, *p* < 0.001; Figure 4D).

### Mediation Analyses

**Figure 5.**
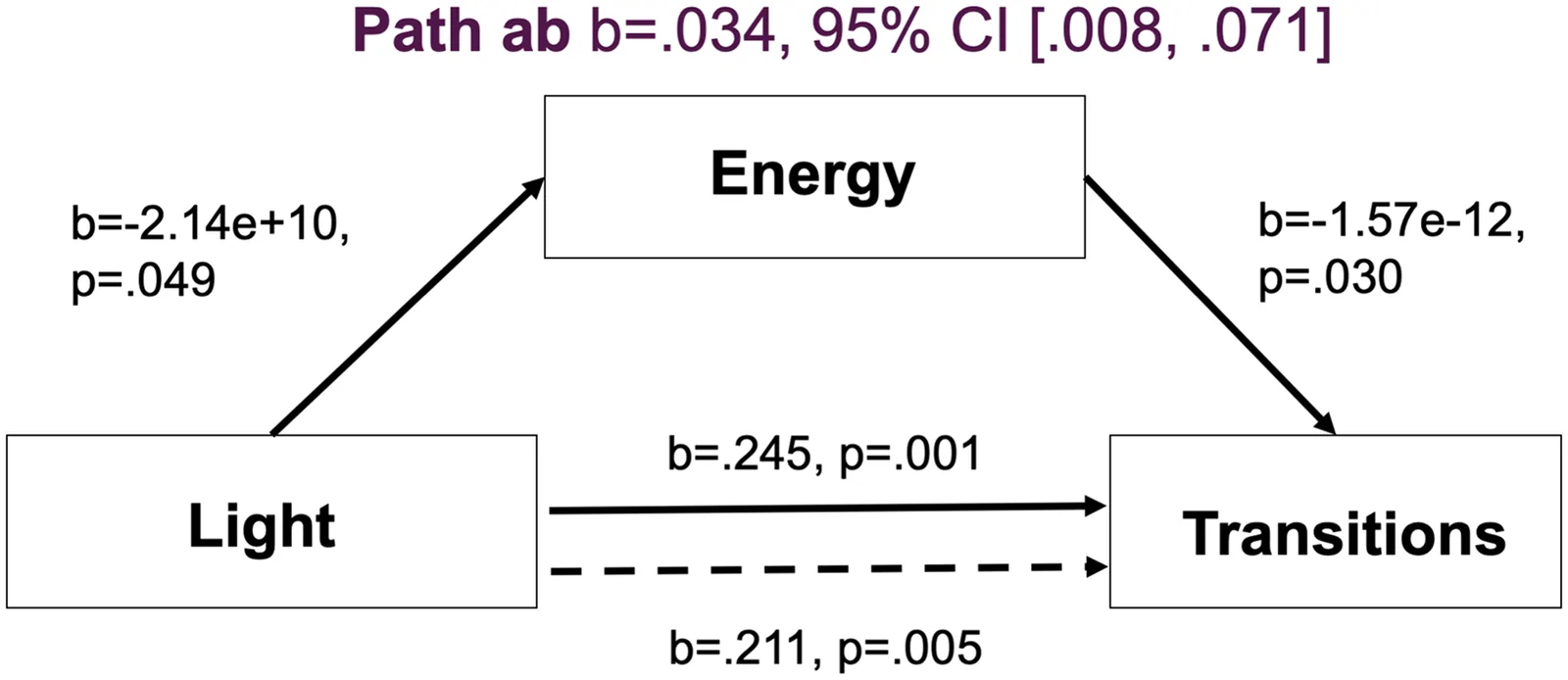
Mediation effects. Mean global control energy partially mediates the effect of light intensity on total transition counts in Model 2.

As in Model 2 (Thalamus_DP_-only control inputs), associations between light intensity, mean global energy, and total transition counts were all significant, we performed mediation analysis. Mean global energy partially mediated the effect of light intensity on total transition counts (total effect: *b* = 0.245, *p* = 0.001; indirect effect: *b* = 0.034, 95% CI [0.008, 0.071]; direct effect: *b* = 0.211, *p* = 0.005; Figure 5).

## DISCUSSION

The current study investigated the effects of light intensity on brain-state transitions and control energy required for those transitions, an aspect that has so far been largely overlooked in efforts to understand the impact of light on brain functions.

Light intensity significantly influenced three brain states: FPN+, a brain state characterized by high activity in the FPN and low activity in the VIS; DMN−, a brain state marked by suppressed activity in the DMN and heightened activity in task-relevant regions, that is, ventral attention network (VAT) and SOM, which encompasses the auditory cortex; and VIS+, a brain state characterized by high activity in the VIS. Prior studies have documented that light luminance affects visual areas, with activity in early visual regions correlating not only with actual illumination levels but also with subjective brightness ratings, reflecting early cortical processing stages (Boyaci et al., 2007; Haynes et al., 2004). Additionally, we observed a reduced occurrence of VIS+ in the second half of light exposure compared to the first half, potentially indicating neural adaptation to sustained light exposure and reduced contributions from rods/cones.

DMN− is a brain state that reflects suppression of DMN and activation of task-positive regions and has been associated with task performance. This state has also been referred to as the task-positive brain state with its occurrence associated with cognitive load of the task and task accuracy (Gu et al., 2021). Similarly, FPN+ is engaged under conditions of high cognitive demand (Cornblath et al., 2020) and its FO has also been associated with improved response time in a visual attentional task induced by methylphenidate (Yan et al., 2025). In youth, FPN+ FO and transitions from DMN to FPN+ increased with age during the *n*-back task indicating functional maturation of the executive control system across development (Cornblath et al., 2020; Satterthwaite et al., 2013). In the current study, we observed a reduction in the occurrence of FPN+ and DMN− states, as well as lower transition probability from DMN− to FPN+ during exposure to high light intensity, while task performance remained stable. The findings suggest that bright light may enhance neural efficiency, possibly optimizing cognitive processing such that the brain may maintain performance with less engagement of high-effort brain states. Interestingly, DMN− and FPN+ occurred more in the second half of light exposure across different light intensities, suggesting a later engagement compared to the VIS and perhaps sustained contribution from ipRGCs. Although not statistically significant, in contrast to FPN+ FO that showed a linear decrease with higher light intensity, DMN− FO showed a slight increase in L3 followed by a significant decrease in L4 that will need to be evaluated in studies with larger samples. Also, it remains unclear whether there is a nonlinear relationship between light intensity and the engagement of the DMN− state (i.e., task positive brain state) (Zeng et al., 2025).

Greater brain variability and more frequent state transitions have been associated with better cognitive performance (Garrett et al., 2011; Nguyen et al., 2017). In contrast, patients with ADHD, bipolar disorder, or opioid use disorder exhibit decreased brain-state transitions and variability that were associated with cognitive impairments (Nguyen et al., 2017; Sun et al., 2021; Ye et al., 2024). Here, we observed more frequent brain-state transitions, suggesting that high light intensity might place the brain in a more adaptable and flexible state. To further understand how light intensity influences brain dynamics and cognitive performance, future research should employ a variety of cognitive tasks across different domains. This would help clarify whether increased brain-state transitions are broadly beneficial or domain-specific.

Regarding control energy, the whole-brain energy demand required for each pairwise brain-state transition may help explain the observed transition probabilities. Under higher light intensity, the control energy required for transitioning from the VIS+ to the FPN− state (characterized by low FPN and high DMN activity) was significantly reduced. The effect of light intensity on transition energy was associated with its effect on transition probability. Additionally, across light conditions, observed brain-state transition probabilities influenced in part by auditory processing and its interaction with light were negatively associated with transition energy, suggesting that control energy underlies brain-state dynamics. Specifically, brain-state transitions that require less control energy are more likely to occur, consistent with previous findings (Gu et al., 2021). To obtain a deeper mechanistic insight of light-induced brain dynamics, we leveraged NCT to explore the role of the visual thalamus. Compared with the model employing uniformly weighted control inputs across the whole brain, the model with dorso-posterior thalamus–only control inputs showed a stronger association between light-induced changes in transition energy and transition probabilities. At the subject level, mean global control energy accounted for the effect of light intensity on total transition counts. These findings are consistent with prior studies highlighting the importance of the dorso-posterior thalamus in mediating the effects of light on the brain (Paparella et al., 2023; Weil et al., 2022). The current study further extends this body of work by demonstrating its potentially functional and metabolic role in shaping light-induced brain-state dynamics. However, relative to the whole-brain control input model, the dorso-posterior thalamus–only model showed weaker associations in the general relationship between transition energy and transition probability. This may reflect the influence of additional processes, such as auditory processing, on observed brain-state dynamics, which likely require control inputs from other brain regions and pathways beyond the dorso-posterior thalamus.

At the regional level, the effects of light intensity on mean control energy for brain-state transitions were at the trend level and did not survive correction for multiple comparisons, possibly due to limited statistical power with a sample size of 20 participants. Nevertheless, the emerging patterns were intriguing and aligned with our previous findings on seasonality. Specially, regions showing a trend toward reduced control energy overlapped with areas previously identified as exhibiting higher metabolic activities in summer including middle frontal gyrus, orbitofrontal, and visual cortices (Zhang et al., 2024). Previous studies have reported a negative association between required control energy and brain glucose metabolism in conditions such as temporal lobe epilepsy and schizophrenia (He et al., 2022; Townsend et al., 2023). Additional regions showing a trend toward reduced control energy included the caudate and insula implicated in decision-making, reward, sensory, and affective processes (Doi et al., 2020; Uddin et al., 2017) as well as the superior temporal gyrus, which plays a key role in auditory processing (Leff et al., 2009). The findings are in line with bright light intervention studies that reported increased striatal processing to risk in a dose-dependent manner (Macoveanu et al., 2016) and decreased functional connectivity within the salience network including insula and superior temporal gyrus after light interventions (Ma et al., 2020). Together, the results suggest that light may facilitate the communications between these regions and other parts of the brain by lowering the energy demands. The observed trend toward increased control energy in the SOM is also noteworthy. In our prior work using longitudinal single-subject fMRI data, stronger within-network resting-state functional connectivity of the SOM was observed in the summer (Zhang et al., 2023). Whether elevated control energy reflects enhanced integration within the SOM and greater functional segregation from other large-scale networks and how this in turn contributes to cognitive and affective functioning remains to be determined. Also, to better understand the biological mechanisms underlying our findings, future studies should directly examine the effects of light intensity on brain glucose metabolism using Fluorodeoxyglucose-positron emission tomography (FDG-PET) in humans.

Several important questions and limitations remain. For example, do the effects of light depend on the brain’s current state, and do they differ depending on whether individuals are engaged in tasks with high versus low cognitive demand? In line with previous findings and our current results, light intensity appears to influence early visual processing. While the current study employed an auditory task to minimize interference from visual stimuli and maintain alertness of participants, it is crucial to investigate how light-induced alterations in early visual processing might affect performance in visually based tasks. Also, the auditory discrimination task used here likely recruited brain regions such as auditory and frontoparietal areas. The engagement of these regions might redistribute neural resources and thereby influence how light intensity affects required control energy and brain-state transitions. Therefore, the current study reveals how light intensity modulates brain-state dynamics under a cognitively engaging condition, which might differ from under a resting condition. Future work comparing resting-state and task-based paradigms under varying light intensities could help disentangle light-specific effects from those resulting from concurrent task engagement. Finally, it would be promising for future research to investigate how individual differences in light sensitivity, which vary by more than 50-fold in healthy individuals as well as altered sensory processing of light, affect brain-state dynamics and control energy under varying light conditions (Phillips et al., 2019; Ríos Llamas et al., 2026). As emerging evidence shows altered light sensitivity and processing in psychiatric populations, such investigations could yield important insights into variability in neural responses to light and their potential clinical relevance (Roguski et al., 2024).

## ACKNOWLEDGMENTS

This work was supported by the National Institutes of Health Pathway to Independence award (K99AA030031, PI: Rui Zhang) and an intramural grant (ZIAAA000550, PI: Nora D. Volkow) from the National Institute on Alcohol Abuse and Alcoholism.

## SUPPORTING INFORMATION

Supporting information for this article is available at https://doi.org/10.1162/NETN.a.586.

## AUTHOR CONTRIBUTIONS

Rui Zhang: Conceptualization; Data curation; Formal analysis; Funding acquisition; Investigation; Methodology; Validation; Visualization; Writing – original draft; Writing – review & editing. Nora Volkow: Funding acquisition; Supervision; Writing – review & editing.

## Funding Information

Rui Zhang, National Institute on Alcohol Abuse and Alcoholism (https://dx.doi.org/10.13039/100000027), Award ID: K99AA030031. Nora Volkow, National Institute on Alcohol Abuse and Alcoholism (https://dx.doi.org/10.13039/100000027), Award ID: ZIAAA000550.

## Supplementary Material



## TECHNICAL TERMS

Brain state: Recurring brain co-activation patterns.
Rods: Retinal photoreceptors that are highly sensitive to low light levels and support vision in dim conditions.
Cones: Retinal photoreceptors responsible for color perception and high spatial acuity under bright light conditions.
Intrinsically photosensitive retinal ganglion cells: Retinal cells containing melanopsin that are most sensitive to blue light and regulate non-imaging forming functions.
Network control theory: A mathematical framework that describes how a complex system such as brain can change from one state to another under structural constraints.
Fractional occupancy: The percentage of TRs (repetition time) assigned to each brain state.
Transition probability: The probability that a brain switches to a different state or remains in the same state (i.e., persistence probability) at the next time point.
Transition energy: The minimum energy required to transition from one brain state to another.
