# Supplementary material for: Effects of light on brain-state dynamics and energy landscape
Source: Netw Neurosci. 2026 Aug 25;10(3):838–52. doi: 10.1162/NETN.a.586 (PMC13569334; doi:10.1162/NETN.a.586)
Supplement: Supplementary file 1 [file netn-10-3-838-s001.pdf]

Supporting information

Effects of light on brain state dynamics and energy landscape

Rui Zhang [a*], Nora D. Volkow [b]

[a] *Department of Psychiatry and Behavioral Neurobiology, University of Alabama at Birmingham, Birmingham, AL 35294, USA*

[b] *National Institute on Drug Abuse, National Institutes of Health, Bethesda, MD 20892, USA*

***Corresponding authors:**

Rui Zhang

ruizhang@uabmc.edu

Figure S1. Order of Light Intensity Conditions and Brain State Transitions by Run and Subject

For each subject and each run (6 minutes, comprising 12 epochs of 30 seconds each, with 3 epochs per light intensity), the order of light stimuli (top row) and the corresponding brain states for each TR (TR = 2 s; bottom row) are shown.

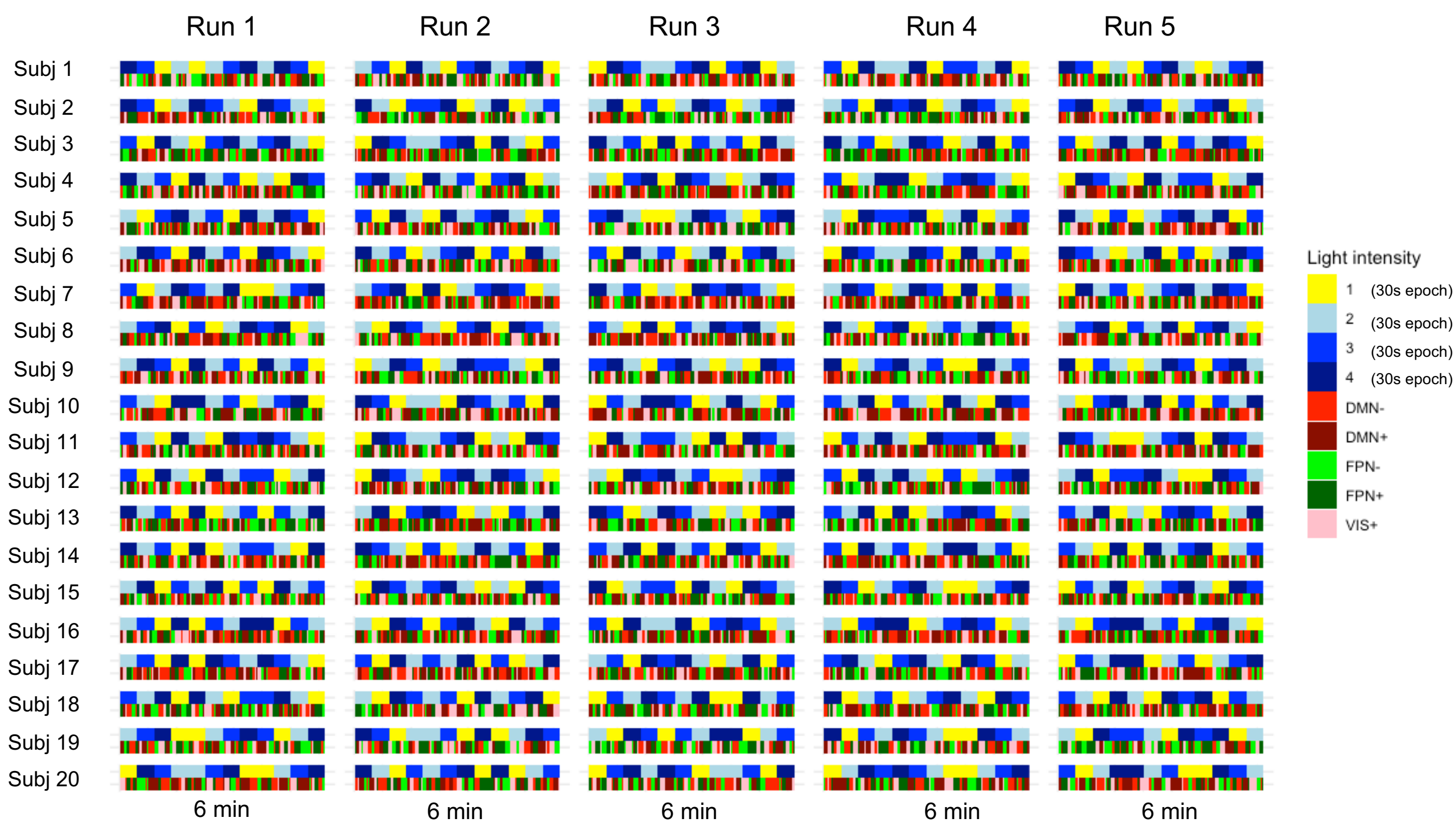

Figure S2 Choosing K
Gained variance explained when k increases from k-1 to k. When k>5, less than 1% of additional variance is explained.

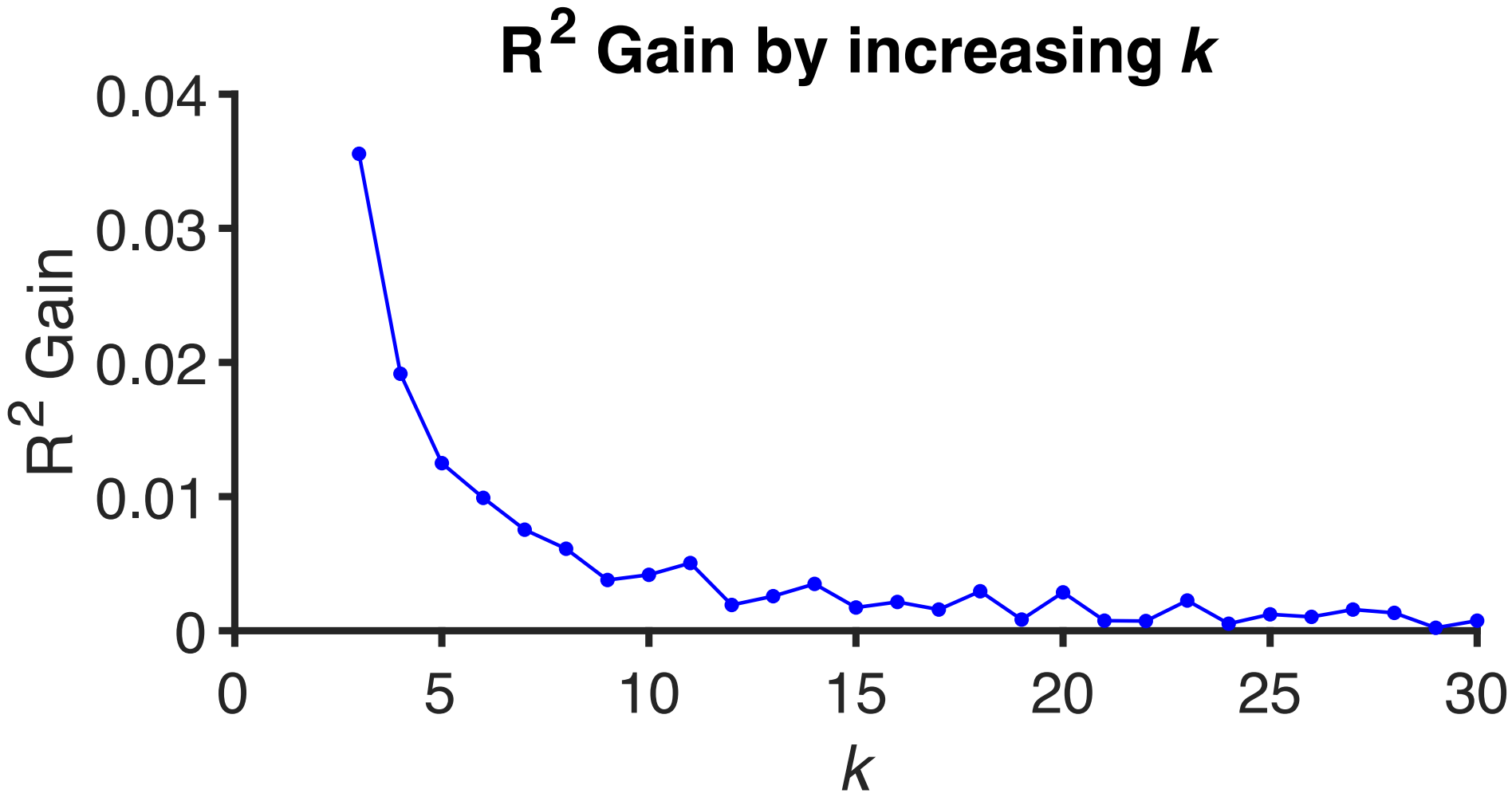

Figure S3 Comparison of brain states in different light conditions
(A) Pearson’s correlations between different brain states under each light intensity condition.
(B) Pearson’s correlations between the centroids of the highest light intensity condition (L4) and of lower light intensity conditions (L1-L3) showing high consistency (diagonal): r=.95-.99

(A)

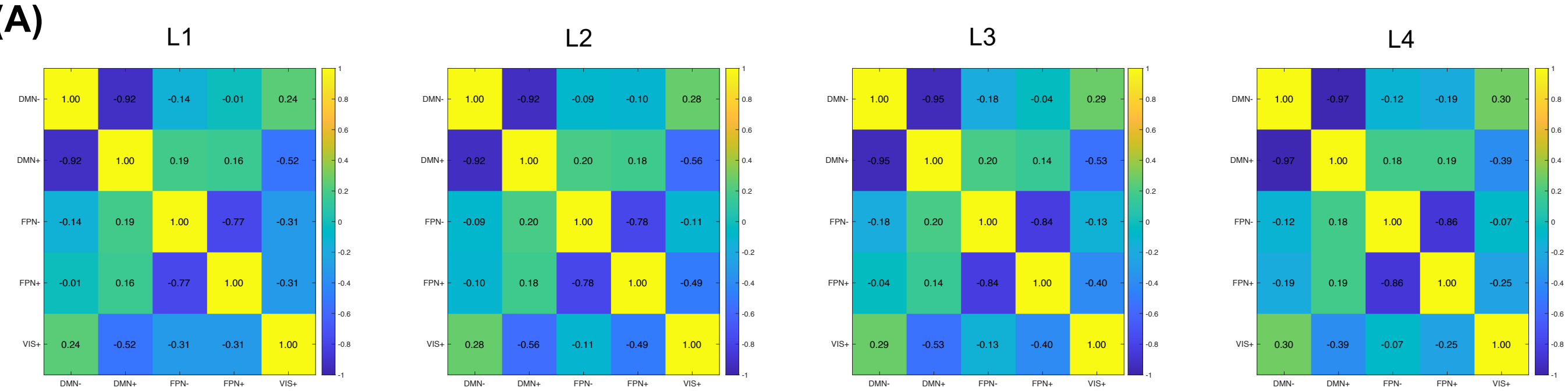

(B)

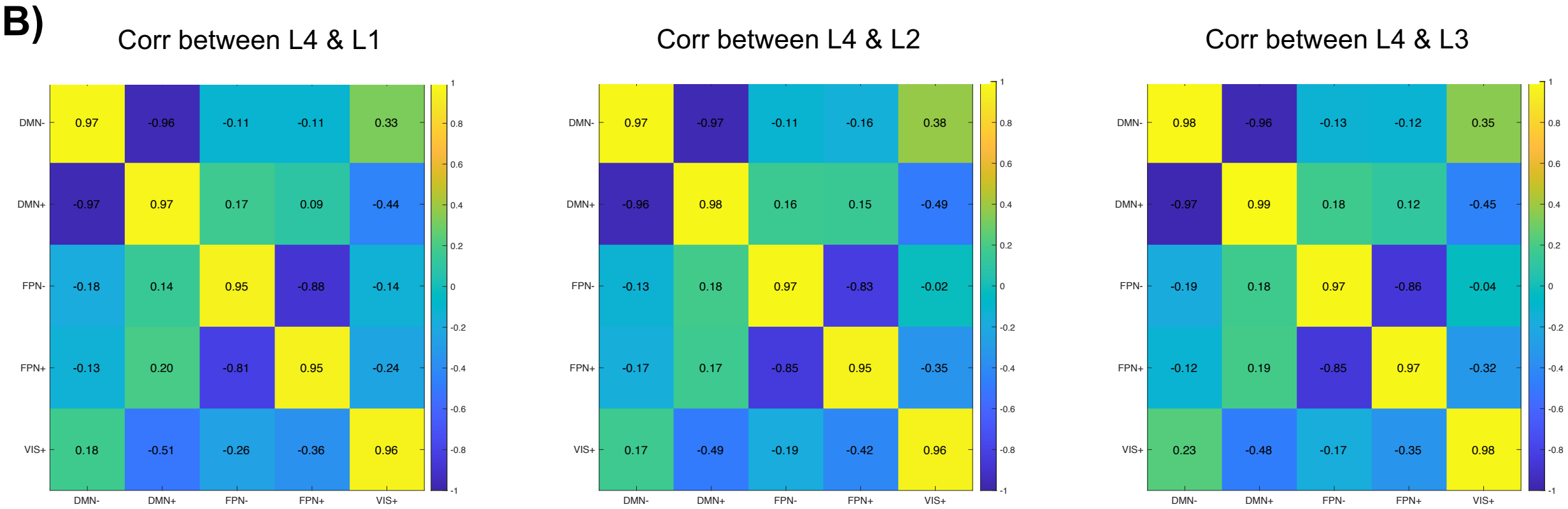

Figure S4 Fractional occupancy of brain states for the first half and the second half of each light intensity

Main effects of light intensity and exposure length were significant. Please see Figure 1 for main effects. No significant interaction between light intensity and exposure length were found. Bars represent the mean, and error bars indicate the standard error of the mean.

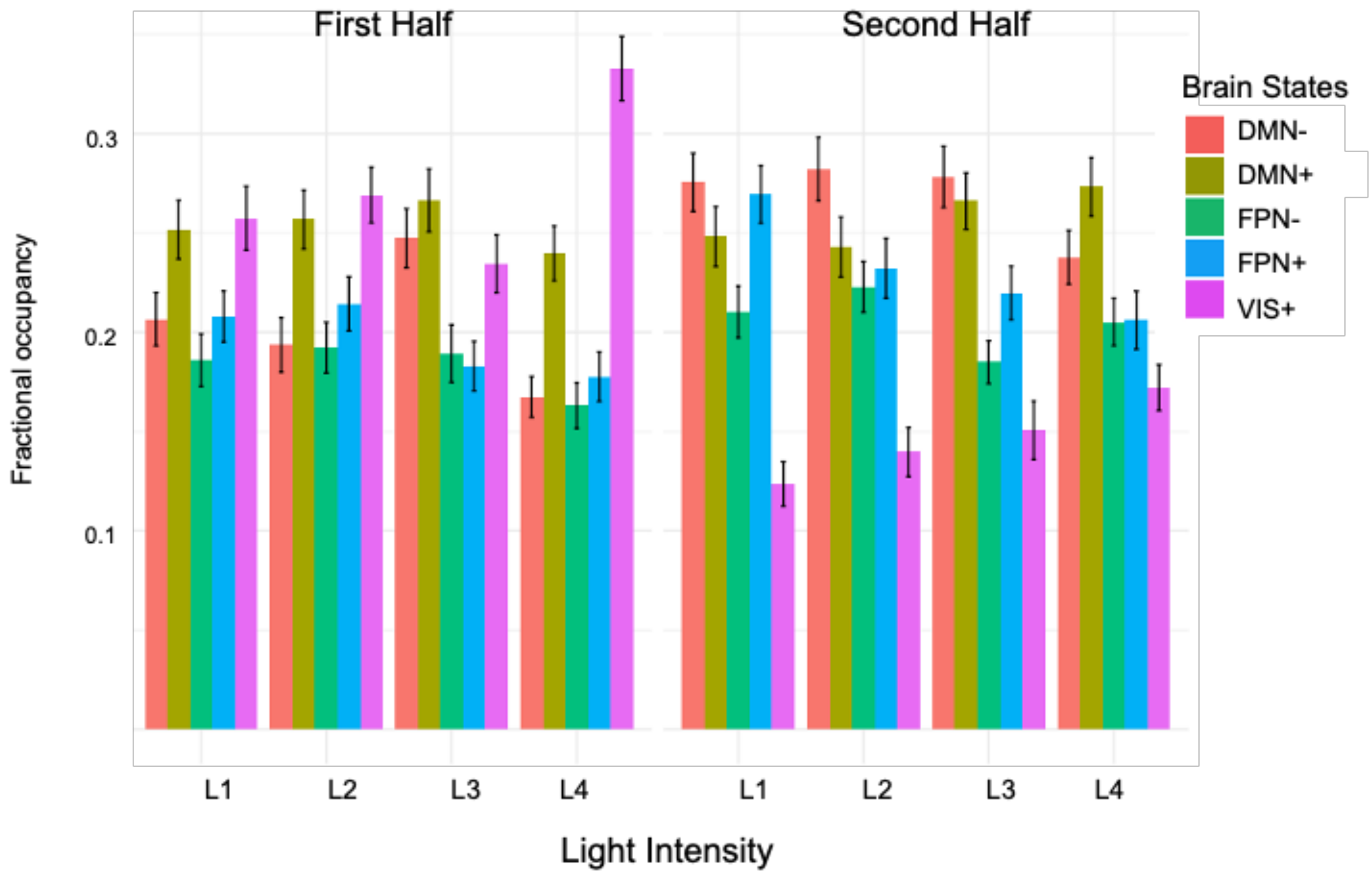

Table S1. Brain Regions Showing Changes in Control Energy as a Function of Light Intensity

ROI names	P_{uncorr}	coefficient	Peak x	Peak y	Peak z	Region names
RH_Vis_19	0.016	-1E-02	9	-74	9	Calcarine
LH_Vis_21	0.002	-1E-02	-7	-74	9	Calcarine
RH_DorsAttn_Post_15	0.028	-1E-02	8	-71	53	Precuneus
RH_Vis_15	0.002	-1E-02	8	-92	-2	Calcarine
LH_Default_pCunPCC_10	0.000	-1E-02	-3	-68	41	Precuneus
LH_Vis_18	0.004	-8E-03	-5	-88	2	Calcarine
RH_Vis_23	0.023	-6E-03	13	-94	19	Occipital Pole
LH_Vis_13	0.047	-4E-03	-13	-43	-5	Lingual
LH_Limbic_OFC_4	0.032	-4E-03	-4	23	-19	Orbitofrontal Gyrus
RH_Cont_PFCl_13	0.007	-4E-03	43	7	51	Middle Frontal Gyrus
RH_Cont_Cing_1	0.040	-3E-03	6	-26	28	Posterior Cingulate Cortex
LH_Cont_Cing_2	0.050	-2E-03	-4	-22	29	Posterior Cingulate Cortex
LH_Default_pCunPCC_4	0.004	-2E-03	-6	-41	24	Posterior Cingulate Cortex
LH_Default_PFC_9	0.048	-2E-03	-6	45	6	Anterior Cingulate Cortex
CAU-tail-lh	0.046	-2E-03	-14	-2	20	Caudate
RH_SalVentAttn_FrOperIns_5	0.045	-2E-03	37	23	5	Insula
LH_SalVentAttn_FrOperIns_5	0.025	-1E-03	-33	19	8	Insula
RH_SalVentAttn_TempOccPar_2	0.037	-1E-03	51	-41	13	Superior Temporal Gyrus
LH_DorsAttn_Post_1	0.048	8E-04	-45	-42	-21	Fusiform
RH_SomMot_7	0.035	2E-03	60	-24	11	Parietal Operculum Cortex
RH_SomMot_27	0.033	2E-03	32	-34	63	postcentral
RH_Vis_7	0.040	2E-03	18	-36	-12	Parahippocampal
RH_SomMot_19	0.046	2E-03	54	-17	40	Postcentral
RH_Limbic_TempPole_7	0.029	2E-03	23	-18	-27	Temporal Pole
RH_SomMot_13	0.045	3E-03	49	-21	19	Parietal Operculum Cortex
RH_SomMot_17	0.030	3E-03	61	-14	30	Postcentral
RH_SomMot_14	0.035	3E-03	62	-12	15	Postcentral
RH_SomMot_23	0.040	4E-03	49	-26	56	Postcentral
